# In-silico and in-vivo evaluation of sesamol and its derivatives for benign prostatic hypertrophy

**DOI:** 10.1007/s13205-021-02952-z

**Published:** 2021-08-14

**Authors:** Abhishek Shah, Aarti Abhishek Shah, Krishnadas Nandakumar, Avinash Kumar, Aravinda Pai, Richard Lobo

**Affiliations:** 1 Department of Pharmacognosy, Manipal College of Pharmaceutical Sciences, Manipal Academy of Higher Education, Manipal, Karnataka 576104 India; 2 Department of Pharmacology, Manipal College of Pharmaceutical Sciences, Manipal Academy of Higher Education, Manipal, Karnataka 576104 India; 3 Department of Pharmaceutical Chemistry, Manipal College of Pharmaceutical Sciences, Manipal Academy for Higher Education, Manipal, Karnataka 576104 India; 4 Present Address: Shobhaben Pratapbhai Patel School of Pharmacy and Technology Management, SVKM’s NMIMS, V.L. Mehta Road, Vile Parle (W), Mumbai, 400056 India

**Keywords:** Benign prostatic hypertrophy, Schrӧdinger, Molecular Modelling, Sesamol, Prostatic index

## Abstract

Pharmacological treatment for BPH includes 5-*α* reductase inhibitors as Finasteride and Dutasteride as a monotherapy or in combination with antimuscarinic drugs, alpha-blockers, 5-phosphodiesterase inhibitor drugs. Androgen receptor inhibitors revealed several adverse events as decreased libido, erectile dysfunction, ejaculatory dysfunction, and gynecomastia. Hence, the emergence of complementary and alternative medications having safety profile—preferably, edible natural products—would be highly desirable. In-silico studies based on Maestro Molecular Modelling platform (version 10.5) by Schrӧdinger, LLC was used to identify the lead molecules. The in-vivo activity studied on rats gave the positive results. The findings based on experiments as antioxidant parameters showed the potential to quench the free radicals. The significant results were also seen in prostatic index and histopathological studies supported the above findings. Based on these data, sesamol and derivative have proven efficacy in protecting against testosterone induced BPH.

**Supplementary Information:**

The online version contains supplementary material available at 10.1007/s13205-021-02952-z.

## Introduction

Benign prostatic hypertrophy (BPH) is an abnormal increase in the size of the prostate gland (Tutolo et al. 2009). Almost all men with average life expectancy are suffered by prostatic disorders. The prevalence of BPH apparent in around 50% of men who are above 50 years, and 90% who are above 80 years (Carson and Rittmaster 2003). The major symptom observed in ageing males is the lower urinary tract symptoms (LUTS) which is a sign of BPH. The reason behind BPH histological condition is obstruction caused in bladder outlet which further obstructs prostate (Mirone et al. 2018).

The interaction between stromal region and epithelial region leads to BPH, androgens being the major influencers of these interactions (Shah et al. 2021). 5-*α*-reductase is well known enzyme for the synthesis of dihydrotestosterone (DHT) (Nieto et al. 2014). DHT is the key player and responsible for producing pathophysiological prostate growth in adult prostate (Shirakawa et al. 2004). Pharmacological treatment for benign prostatic hypertrophy (BPH) is either monotherapy of 5-*α* reductase inhibitors or a combination of antimuscarinic drugs, *α*-blockers, 5-phosphodiesterase inhibitors with 5-*α* reductase inhibitors. 5-*α* reductase inhibitors (ARI’s) revealed several adverse events as decreased libido, erectile dysfunction, ejaculatory dysfunction, and gynecomastia (Trost et al. 2013). Hence, the emergence of complementary and alternative medications having safety profile (Shah et al. 2019)—preferably, edible natural products- would be highly desirable (Keehn and Lowe 2015). Hence, this experimental study was designed and executed to investigate the potential of sesamol (SM) and 3′,4′-(Methylenedioxy) acetophenone (3′MA) on testosterone-induced experimental BPH models in vivo.

## Materials and methods

Maestro Molecular Modelling platform (version 10.5) by Schrӧdinger, LLC used for the Molecular docking studies on 3,4-(Methylenedioxy) phenol and its derivatives (Table 1).

**Table 1:** The ligands considered for study

Sr. no.	Sesamol and its derivatives	Synonyms	Structure
1	Sesamol (PubChem CID: 68289)	3,4-(Methylenedioxy)phenol, 5-Benzodioxolol	
2	Piperonylic acid (PubChem CID: 5370536)	1,3–Benzodioxole–5–carboxylic acid, 3,4-(Methylenedioxy) benzoic acid	
3	3,4-(Methylenedioxy) benzyl alcohol (PubChem CID: 10322)	1,3–Benzodioxole–5–methanol, 3,4-(Methylenedioxy) phenyl methanol Piperonyl alcohol	
4	Piperonyl amine (PubChem CID: 75799)	1,3–Benzodioxole–5–methylamine, 3,4-(Methylenedioxy) benzylamine	
5	3,4–(Methylenedioxy)–cinnamic acid (PubChem CID: 643181)	(E)–3–(1,3–benzodioxol–5–yl)prop–2–enoic acid	
6	3′, 4′-Methylendioxy acetophenone (PubChem CID: 76622)	3′,4′–(Methylenedioxy)acetophenone 1–(Benzo[*d*][1,3]dioxol–5–yl)ethanone Acetopiperone 5–Acetyl–1,3–benzodioxole	
7	3,4-(Methylendioxy) phenylacetic acid (PubChem CID: 76115)	1,3–Benzodioxole–5–acetic acid, Homopiperonylic acid	
8	3,4-(Methylenedioxy) mandelic acid (PubChem CID: 119618)	*α*–Hydroxy–1, 3–benzodioxole–5–acetic acid	
9	3,4-(Methylenedioxy) aniline (PubChem CID: 84310)	5–Amino–1,3–benzodioxole	

The atomic coordinates of the androgen receptor were downloaded from the Protein Data Bank using PDB ID 2AMA (Pereira de Jésus‐Tran et al. 2006) Eight analogues of 3,4-(Methylenedioxy) phenol were identified and further screened for their binding affinity to the androgen receptor.

### Ligand preparation

Lowest energy 3D structures with corrected chiralities were produced by ligand optimization using the tool LigPrep tool (Sastry et al. 2013). OPLS 2005 force field used and the process performed at neutral pH.

### Protein preparation and grid generation

Before the docking study, the biological unit of protein subjected to the protein preparation where it added with side chains and missing residues by Prime tool. Refining of the protein done by heavy atom and water molecule removal in restrained optimization. After the protein preparation, the receptor grid was generated using the OPLS 2005 (Shivakumar et al. 2010). The cubic box with specific dimensions generated around the centroid of the active site.

### Ligand docking

The standard precision (SP) and extra precision (XP) flexible glide docking were used to screen the analogues, using the Glide tool. For ligand atoms, Van der Waals factor and partial charge cut off were selected to be 0.80 and 0.15, respectively, and ligands that are optimised used for this purpose. Root mean square deviation (RMSD) calculations performed on the bound ligand. The lowest glide sore with the best dock poses recorded for each ligand.

### Induce fit docking

Induced fit docking (IFD) allows for more protein flexibility than the standard SP or XP docking protocols. It starts with the docking of ligands employing Glide for generating  binding poses for active ligands. IFD employs reduced Van der Waals radii and the cut-off for coulomb-vdW is increased. During docking, highly flexible side chains can be removed temporarily and then for each pose generated, ligands are accommodated by re-orientation of closely placed side chains. This step in IFD employs Prime module. Finally, redocking of each ligand is performed by SP docking protocol.

### ADME studies

ADME studies were performed employing QikProp tool of Maestro suite. QikProp module calculates many property descriptors like molecular weight, partition co-efficient, hydrogen bond donor, hydrogen bond acceptor, percentage oral absorption, cardiac toxicity, polar surface area, Lipinski’s rule of five etc. Based on these parameters the drug-likeness of the compounds were predicted.

### Molecular dynamics simulations

Molecular dynamics (MD) simulations study involves three steps viz. system builder, minimization and molecular dynamics simulation. In the first step, the complex of ligand and protein was solvated using SPC as the solvent and orthorhombic box. Charges were neutralised by adding chloride and sodium ions. The generated system was energy minimised in the second step by employing OPLS3e force field. Finally, the minimised complex was put for simulation for a total period of 20 ns and a frame was captured every 20 ps.

### In-vivo efficacy model

Testosterone (Cernos Depot 1Gm Injection) was purchased from Sun Pharmaceutical. Sesamol and 3′,4′-(Methylenedioxy) acetophenone were purchased from Sigma-Aldrich. All the analytical grade chemicals were procured from Merck Limited, India and Hi-media. Plastic labware and syringes were procured from tarson and BD biosciences, respectively.

#### Animals and experimental procedures

The study was conducted in compliance with Committee for the Purpose of Control and Supervision of Experiments on Animals (CPCSEA) guidelines with the prior approval of Institutional Animal Ethical Committee at Central Animal Research Facility, Manipal) [Approved reference number—IAEC/KMC/04/2016]. Adult male Wistar rats (approx. 180–200 g) were used for conducting the experiments.

#### Induction of BPH in rats by *T* and the protective effect of SM and 3′ MA on it

BPH was induced by orchiectomy/castration surgical procedure by a scrotal incision using thiopentone sodium (30 mg/kg i.p) as an anaesthetic. Castration surgery was performed to remove the effect of endogenous testosterone. After induction of anaesthesia, the scrotum was incised to expose the testicles and the epididymis, and the spermatic cord and both of testes were removed. The incised part was sutured (Xu et al. 2014). After castration, the rats were kept for seven days to induce hyperplasic growth of the prostatic gland (Table 2). BPH was induced in the animals by the administration of testosterone undecanoate (TU) (3 mg/kg/7 days/week/s.c) in olive oil for 28 days (Mitra et al. 1999). The animals were treated with SM (50 and 100 mg/kg p.o) and 3′MA (50 and 100 mg/kg p.o) suspended in carboxyl methylcellulose (CMC) (0.25%) throughout the study. According to the reported literature, SM at the dose of 50 mg/kg delayed wound healing (Shenoy et al. 2011) while 100 mg/kg was found to be effective in dinitrochlorobenzene-induced inflammatory bowel disorder. Hemalatha et al. (2013) reported that SM (50, 100 and 200 mg/kg p.o) showed promising effects on deoxycorticosterone acetate–salt-induced oxidative stress in hypertensive rats. Therefore, SM at the dose of 50 and 100 mg/kg was selected for the present study. 3′MA were also investigated in the same dose range for the comparative analysis. Finasteride (1 mg/kg p.o)(Kim SK, Seok H, Park HJ, Jeon HS, Kang SW, Lee BC, Yi J, Song SY, Lee SH, Kim YO 2015) was used as a standard drug for the study. After 24 hr of the last s.c. injection, rats were sacrificed. Prostate tissues were removed and weighed. One section of the ventral lobe of the prostate gland was fixed in 10% formalin for histology. Another section of the prostate was kept in Tris-HCl buffer, pH 7 and stored at − 80 °C till further antioxidant analysis.

**Table 2:** Animal grouping and treatment

Experimental group	Treatment (*n* = 6) for 28 days
Normal control (NC)	No castration vehicle olive oil (i.p) + 0.25% CMC (p.o)
Sham	Surgery performed (intact testes) vehicle olive oil (i.p) + 0.25% CMC (p.o)
Disease control (DC)	Castrated animals treated with testosterone undecanoate (TU) (3 mg/kg/day; s.c) in olive oil
SM 50	Castrated animals treated with TU + SM (50 mg/kg p.o in 0.25% CMC)
SM 100	Castrated animals treated with TU + SM (100 mg/kg p.o in 0.25% CMC)
3′ MA 50	Castrated animals treated with TU + 3′MA (50 mg/kg p.o in 0.25% CMC)
3′ MA 100	Castrated animals treated with TU + 3′MA (100 mg/kg p.o in 0.25% CMC)
Finasteride (Fina 1)	Castrated animals treated with TU + Standard drug Finasteride (1 mg/kg p.o in 0.25% CMC)

#### Prostatic index (PI)

Prostate tissues were harvested and weighed instantly then prostate index was calculated as the ratio of the prostate weight to the total body weight (Mosli et al. 2015):

$${\text{PI }} = \, \left( {{\text{prostate weight}}/{\text{body weight}}} \right) \, \times { 1}00.$$

#### Histopathological examination

The prostate tissues were fixed and embedded in paraffin wax followed by sectioning. The thin sections were stained using haematoxylin and eosin. The stained sections were mounted on a glass slide and are observed under an inverted microscope and images were taken.

#### Determination of antioxidant parameters

Antioxidant assays of ventral prostate homogenate {Catalase, Lipid Peroxidation, Nitrite, GSH} were screened.

##### Prostate homogenate preparation

Ventral prostate tissue was homogenised in ice-cold 0.1 mol/L of Tris-HCl buffer, pH 7.4, and centrifuged at 3000 rpm for 15 min. The supernatant was collected and used for antioxidant assay estimations.

- Total protein estimation
  The total protein content was measured using thermo-scientific pierce bicinchoninic acid (BCA) protein assay kit.
- Total glutathione (GSH) (Rahman et al. 2007)
  GSH estimation was performed as per previous literature. The final-coloured product (TNB) was quantified at 405 nm in an ELx808 absorbance microplate reader (Bio-Tek, USA).
- Estimation of catalase activity (Weydert and Cullen 2010)
  Catalase enzyme activity was determined by the procedure as per reported studies. Catalase unit was defined by decomposition of hydrogen peroxide/min at room temperature and neutral pH.
- Lipid peroxidation assay (TBARS) (Garcia et al. 2005)
  Polyunsaturated fatty acids is converted to malondialdehyde (MDA) on decomposition reaction. The product can determine the extent of the peroxidation reaction. Malondialdehyde in the presence of thiobarbituric acid (TBA) develops a pink colour which was read at 532 nm.
- Nitrite assay (Miranda et al. 2001)

Nitrite assay was performed using Griess reagent a as per reported literature. Griess reagent is composed of Naphthyl Ethylene Diethylamine Dihydrochloride (NEDD) and Sulfanilamide. {NEDD—0.1% (*w*/*v*) in 5% (*v*/*v*) orthophosphoric acid H_3_PO_4_ and sulfanilamide 1% (*w*/*v*) in 5% (*v*/*v*) Orthophosphoric acid H_3_PO_4_. To 100 µL of the prostate homogenate, 100 µL of Griess reagent added. The absorbance was measured at 540 nm after 10 min of incubation in dark condition.

### Statistical analysis

The data were analysed statistically and expressed as mean ± SEM (*n* = 6). Groups were compared using One-way ANOVA followed by Dunnett’s post hoc test for multiple comparisons. The level of significance set at *p* < 0.05.

## Results

### Human androgen receptor – 2AMA

**Fig. 1:**
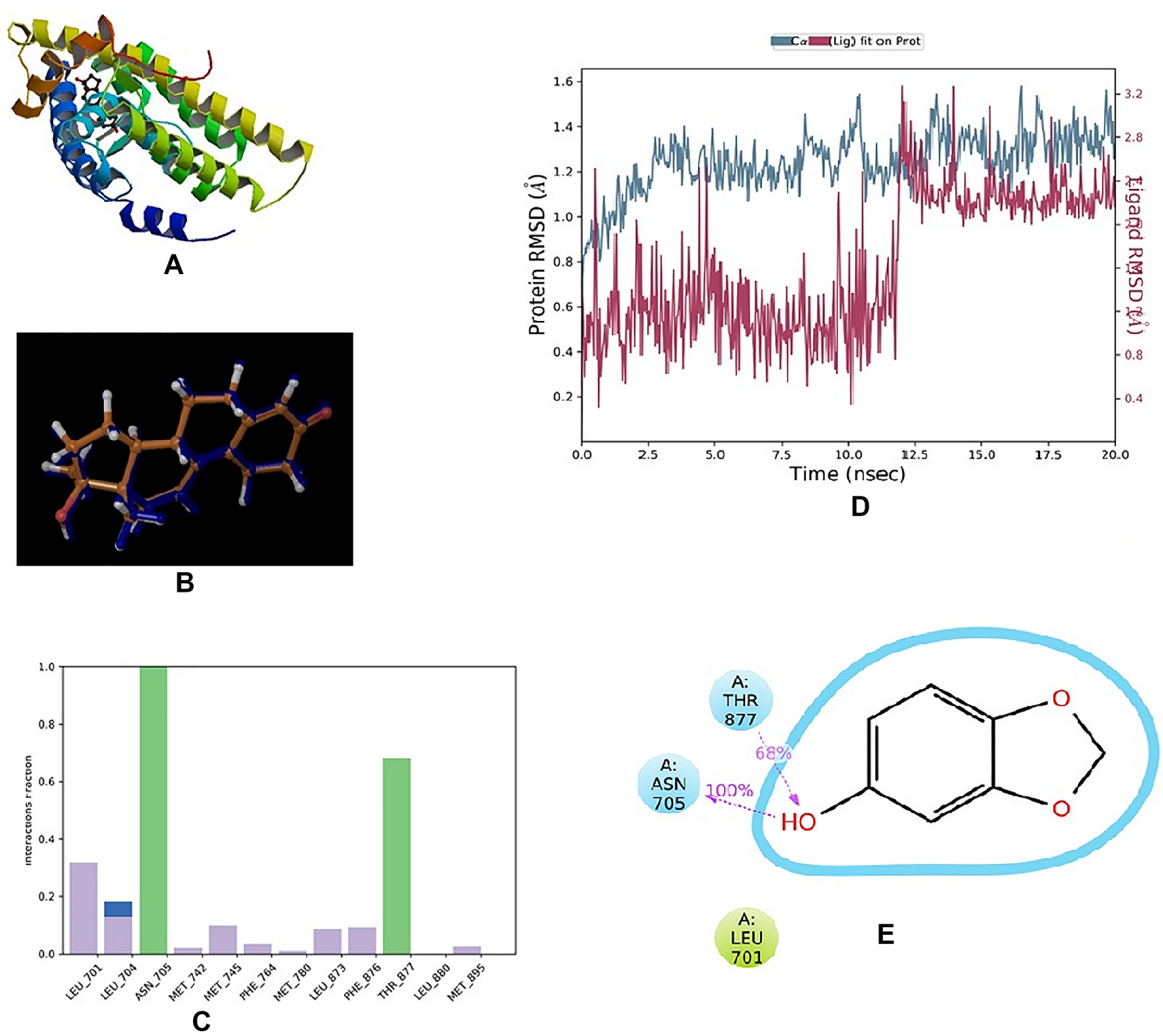
**A** Human Androgen Receptor—1E3G; **B** Dock ligand and ligand superposition RSMD Calculation—0.1236; **C** Histogram; **D** RMSD plot for SM and Human androgen receptor; **E** Ligand–protein 2-D interaction diagram

Figure 1A and B showing the 3D-crystal structure of 2AMA which was downloaded from PDB. The results of Docking were noted and reported in Supplementary Table 1. QikProp tool from the Mestro suit was used to calculate the ADME and the results are shown in Supplementary Table 2

### Ligand interaction

Docking experiments were performed with SP and XP type. The ligand interaction diagrams were captured for each ligand for XP docking and are shown in Supplementary Fig. 1.

### MD simulation

This experiment was performed with the Desmond and the following two outputs were observed.

#### RMSD plot for SM and human androgen receptor

The plot was obtained and reported as Fig. 1D

#### Histogram of protein–ligand contact

The histogram was reported in Fig. 1C and the ligand interactions with amino acids were captured and shown in Fig. 1E.

### In-vivo efficacy

#### Prostatic index

**Fig. 2:**
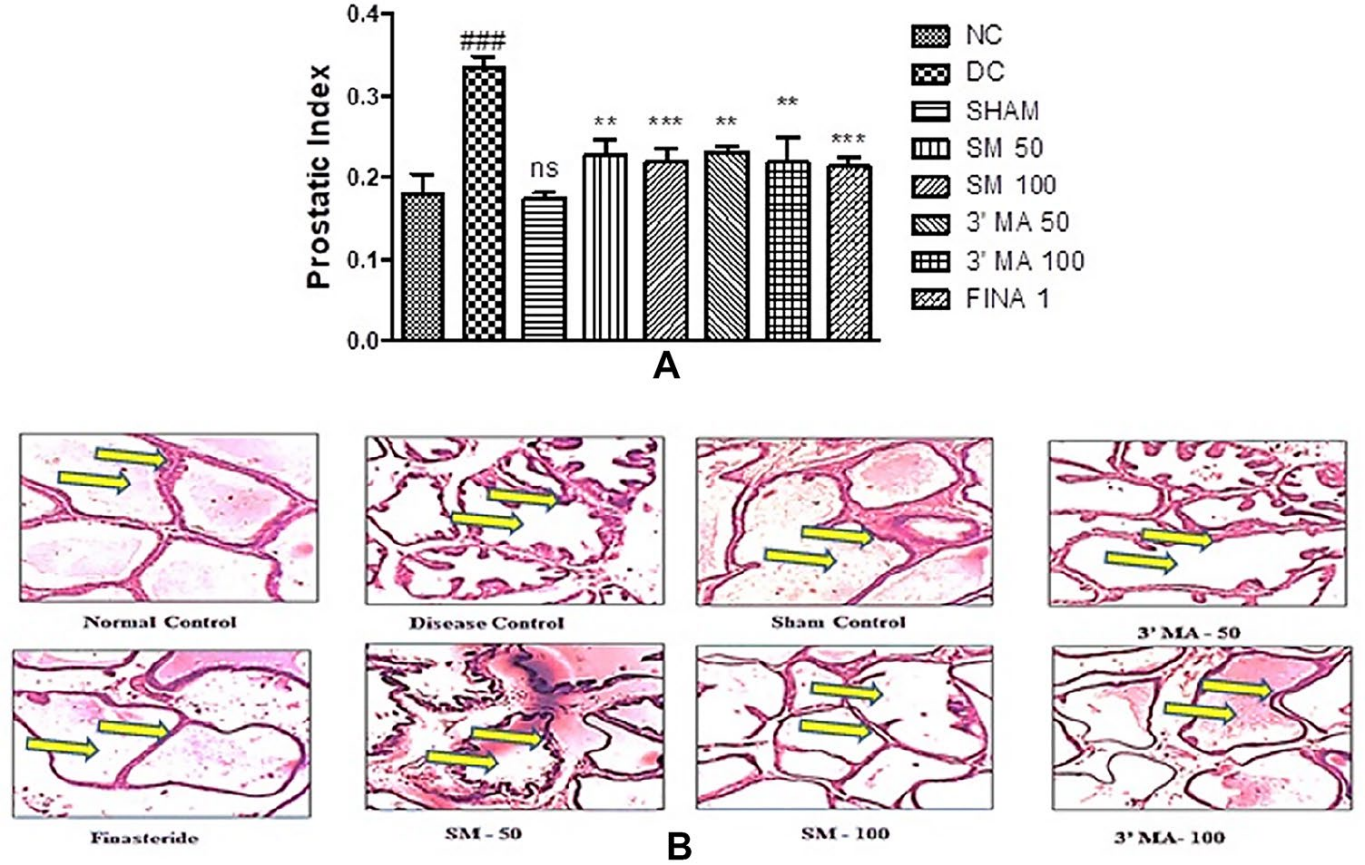
**A** Effect of SM and 3′MA treatment in two different doses (50 mg/kg, 100 mg/kg, orally, for 28 days) on the prostatic index. #Represents significant difference in means in comparison to normal control (NC) group data with the disease control (DC) and sham group (Sham); while * indicates notable variation in means when compared to DC group with the test drug treatment groups. ns implies non-significant; **B** Histological examination of rat ventral prostates (100 × images)

In the testosterone-treated animals, (3 mg/kg) there was significant increase in prostatic index (53.82%) in comparison with Control group). The prostatic index SM 50 and 100 mg/kg/day for 28 days by p.o. (along with testosterone) was 67.91% and 65.67% and found significant in prevention of an increase in the prostatic index as compared with the disease control group. In case of 3′MA, at the dose of 50 and 100 mg/kg, the prostatic index was reduced to 68.98% and 65.34% which is comparable to the standard drug, finasteride (% reduction is 63.73) Fig. 2A

#### Effect of SM and 3′MA treatment on antioxidant status of ventral prostate

Effect of SM and 3′MA treatment was studied at doses (50 and 100 mg/kg, p.o., 28 days) on-antioxidant markers as nitrite, lipid peroxidation, catalase, and GSH level were evaluated and quantified as mentioned in Supplementary Table 3.

#### Histopathological examination

The histopathological evaluation represents the morphological changes, predominantly in the epithelial lining of the prostate gland. In normal control and sham group, the normal lining epithelial cells were indicated the stress induced by castration surgery did not influence the histological changes in the prostate gland. In the prostate section of a diseases control group, testosterone treatment-induced prostatic hypertrophy along with epithelial lining has been observed with broader thickness and formation of polyps’. The structural alteration represents the induction of BPH. Testosterone groups co-treated with SM and 3′MA (50 and 100 mg/kg) showed a marked decrease in hypertrophy and hyperplasia with recovery in the epithelial lining in a dose-dependent manner (Fig. 2B).

## Discussion

The in-silico studies also supported our findings that SM and 3′MA have shown potential in preventing the induction of BPH by testosterone. Molecular docking (SP and XP), as well as induced fit docking, suggests that, the screened compounds might bind with the human androgen receptor as the docking score and IFD score was more than − 6.0 and − 500.0, respectively. The  MD analysis for SM suggests that the protein-SM complex was stable throughout the simulation period as RMSD value was observed to be less than 3 Å Although there was drift for the initial 12.5 ns the complex stabilised afterwards till the end. SM showed H-bond interaction with ASN 705 and THR 877 amino acid residues. ADME analysis by QikProp tool also suggests that these compounds have an acceptable pharmacokinetic profile.

The hyperplastic cell growth and impaired proliferation of cells can be caused by oxidative stress (Udensi and Tchounwou 2016). The data from BPH patients reflects an increased Glutathione S-transferases (GST) activity and malondialdehyde (MDA) levels. These findings from the literature suggest that, there is a generation of free radicals and  role of antioxidants in BPH patients. Nitric oxide synthase (NOs) (El Rassy et al. 2017); peroxides were observed to be raised BPH patients. On the other hand, levels of superoxide dismutase were decreased and the same trend was observed for catalase in BPH (Aryal et al. 2007). Hence, a correlation between BPH and oxidative stress was studied and reported in the literature as evidence (Gangemi et al. 2014).

Several natural compounds and dietary polyphenols including SM have gained attention due to their antioxidant potential. Still, the management of BPH by dietary supplements is under investigation and need to be explored as a preventive treatment option in BPH (Eleazu et al. 2017).

The antioxidant potential of SM and its beneficial pharmacological activities were reported in the literature. Geetha et al. in vitro studies reported that SM at a variety of doses and test systems such as H_2_O_2_ assay, DPPH assay, NO scavenging, Lipid peroxidation in brain and liver showed a wide spectrum of antioxidant activity (Geetha et al. 2009). SM proficiently quenched hydroxyl, lipid peroxyl, one-electron oxidising and tryptophanyl radicals. SM studied in biochemical experiments revealed that it inhibits lipid peroxidation, RNA and DNA degradation. In the Solvation Model Based on Density (SMD) continuum model, SM shows the exceptional potential of peroxyl radical quenching in an aqueous medium under physiological conditions (Galano et al. 2011). SM protects against the organ damage by reducing nitric oxide-associated lipid peroxidation in endotoxin-treated rats (Chu et al. 2006).

Therefore, in our present study, the antioxidant potential of SM and 3′MA was explored in BPH. In this study, rats treated with SM and 3′MA showed a significant reduction in prostatic index comparison to the disease control group (testosterone-treated). The change in the prostatic index was further confirmed with antioxidant assays and histological examination.

In antioxidant studies, SM and 3′MA treatment inhibited the level of malondialdehyde (MDA) and H_2_O_2_ in lipid peroxidation and catalase assay. The effect may be due to its scavenging properties of free radicals and thereby gave an insight into the protective mechanism.

The significant increase in GSH levels in SM and 3′MA treated groups while a decrease in contents of nitrite was observed. (Supplementary Table 3). The enhanced oxidative capacity observed in SM and 3′MA treated groups can be said to the credible mechanism as the antioxidant potential to prevent BPH.

There were no alterations in the histological examinations of rat tissue of prostate in the NC- and Sham groups. The sections of tissues showed cuboidal epithelial cells with intact lining.

The development of BPH, in the disease control group, was confirmed by hyperplastic cells which further justified with damaged linings of epithelium and cytoplasm with many vacuoles. Also, there was shrinkage in glandular luminal area. In SM and 3′MA  treated rat  doses as 50 and 100 mg/kg was observed to prevent hyperplasia and confirmed by the observation that tissue slides showed the same type of cells as observed in the control group. SM and 3′MA at 100 mg/kg dose showed marked recovery in the prostatic histoarchitecture especially in epithelial cell lining as compared to 50 mg/kg dose. (Fig. 2B) This histopathology observations supports the prophylactic potential against BPH for tested compounds. The standard group treated with finasteride prevented hyperplastic growth.

The above finding reveals the antioxidant potential of SM might be the key mechanism to prevent BPH.

## Conclusion

Based on these data, SM and 3′MA have shown potential in  prevention of BPH induced by testosterone. In conclusion, SM and 3′MA are  the promising candidates for further investigation in prostatic disorders like prostate cancer and clinical trials.

## Supplementary Information

Below is the link to the electronic supplementary material.

Supplementary file1 (DOCX 138 KB)
