# Supplementary material for: In-silico and in-vivo evaluation of sesamol and its derivatives for benign prostatic hypertrophy
Source: 3 Biotech. 2021 Aug 14;11(9):411. doi: 10.1007/s13205-021-02952-z (PMC8364611; doi:10.1007/s13205-021-02952-z)
Supplement: Supplementary file 1 — Supplementary file1 (DOCX 138 KB) [file 13205_2021_2952_MOESM1_ESM.docx]

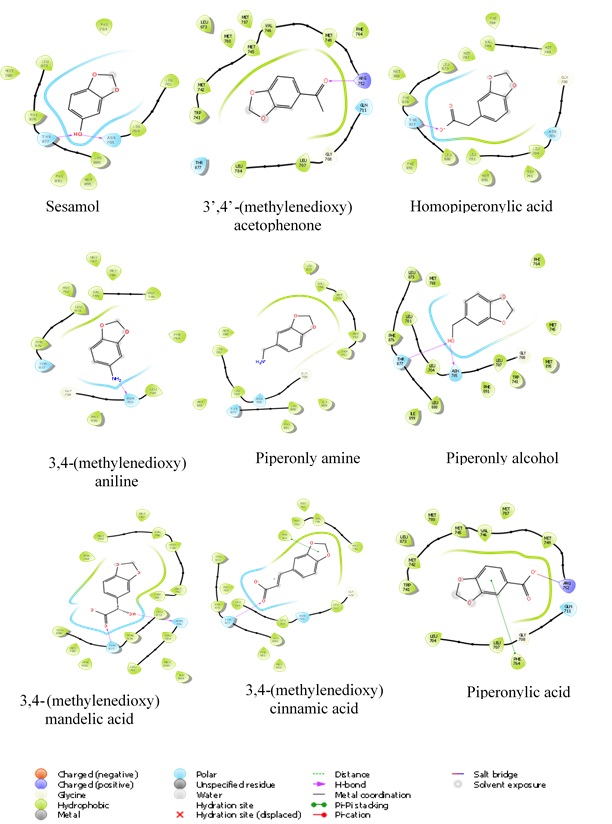
**Supplementary Figure 1 Ligand interaction**

**Supplementary Table 1. Ligand docking and Induced fit docking (IFD):**

| Sr. No. | Ligands | SP Score | XP Score | IFD Score | BBB penetration by Swiss ADME study |
| --- | --- | --- | --- | --- | --- |
| 1 | Homopiperonylic acid | -7.185 | -6.746 | -523.543 | Yes |
| 2 | 3',4'-(Methylenedioxy) acetophenone | -6.931 | -6.508 | -524.327 | Yes |
| 3 | 3,4-(Methylenedioxy) mandelic acid | -6.661 | -7.327 | -522.806 | No |
| 4 | Piperonylic acid | -6.618 | -6.352 | -522.781 | Yes |
| 5 | Piperonly alcohol | -8.036 | -7.182 | -524.477 | Yes |
| 6 | Sesamol | -7.514 | -6.67 | -523.119 | Yes |
| 7 | 3,4-(Methylendioxy)-cinnamic acid | -6.162 | -7.051 | -522.818 | Yes |
| 8 | 3,4-(Methylenedioxy) aniline | -7.232 | -6.761 | -523.243 | Yes |
| 9 | Piperonly amine | -6.823 | -6.841 | -522.81 | Yes |
| 10 | Finasteride | -7.528 | -8.415 | -527.36 | Yes |

**Supplementary Table 2: ADME Analysis**

| Sr. No. | Ligands | Mol weight | QPlogPo/w | QPlogS | QPPCaco | Rule of Five | % of Human Oral Absorption |
| --- | --- | --- | --- | --- | --- | --- | --- |
| 1 | Sesamol | 138.1 | 0.926 | -0.721 | 3005.765 | 0 | 94.617 |
| 2 | Piperonylic acid | 166.1 | 0.976 | -0.852 | 253.962 | 0 | 75.7 |
| 3 | Piperonly alcohol | 152.1 | 0.92 | -0.777 | 3028.362 | 0 | 94.639 |
| 4 | Piperonly amine | 151.1 | 0.42 | 0.315 | 647.345 | 0 | 79.717 |
| 5 | 3,4-(Methylendioxy)-cinnamic | 192.1 | 1.542 | -1.482 | 203.786 | 0 | 77.305 |
| 6 | 3',4'-(Methylenedioxy) acetophenone | 164.1 | 1.042 | -0.644 | 3158.204 | 0 | 95.683 |
| 7 | Homopiperonylic acid | 180.1 | 1.334 | -1.198 | 280.697 | 0 | 78.576 |
| 8 | 3,4-(Methylenedioxy) aniline | 137.1 | 0.505 | -0.619 | 2577.923 | 0 | 90.959 |
| 9 | 3,4-(Methylenedioxy) mandelic acid | 196.1 | 0.534 | -0.954 | 120.024 | 0 | 67.285 |

**Supplementary Table 3: Quantitative estimation of Antioxidant parameters in prostate tissue homogenate**

| Animal Groups | Nitrite (nmol of CAT/mg protein) | Lipid peroxidation (nmol of  MDA formed/mg protein | CAT (nmol of CAT/mg protein) | GSH (nmol of GSH/mg protein) |
| --- | --- | --- | --- | --- |
| NC | 728.1 ± 75.2 | 71.14±4.53 | 1.76 ± 0.17 | 1.961±0.1568 |
| DC | 2102.0 ± 84.9^###^ | 114±4.716^###^ | 0.61± 0.13^#^ | 0.637±0.0587^###^ |
| Sham | 953.3 ± 190.6^ns^ | 69.94±6.048 ^ns^ | 1.81 ± 0.20 ^ns^ | 2.48±0.2008 ^ns^ |
| SM-50 | 578.0 ± 151.0^***^ | 71.12±4.529^***^ | 2.60 ± 0.53^**^ | 2.403±0.1739^***^ |
| SM-100 | 678.5 ± 172.5^***^ | 54.62±4.45^***^ | 1.45 ± 0.31 ^ns^ | 2.152±0.2506^***^ |
| 3’MA 50 | 606.0 ± 90.5^***^ | 64.39±6.6^***^ | 1.93 ± 0.45^*^ | 2.222±0.2257^***^ |
| 3’MA 100 | 878.2 ± 111.8^***^ | 62.82±3.669^***^ | 1.45 ± 0.21 ^ns^ | 2.341±0.08276^***^ |
| Finasteride | 736.0 ± 74.6^***^ | 65.06±2.889^***^ | 1.89 ± 0.13^*^ | 2.601±0.347^***^ |
| # Represents significant difference in means in comparison to normal control (NC) group data with the disease control (DC) and sham group (Sham); while * indicates notable variation in means when compared to DC group with the test drug treatment groups. ns implies non-significant | | | | |
